# The Impact of the Biological Variability or Assay Performance on AMH Measurements: A Prospective Cohort Study With AMH Tested on Three Analytical Assay-Platforms

**DOI:** 10.3389/fendo.2018.00603

**Published:** 2018-10-16

**Authors:** Leif Bungum, Julia Tagevi, Ligita Jokubkiene, Mona Bungum, Aleksander Giwercman, Nick Macklon, Claus Yding Andersen, Tobias Wirenfeldt Klausen, Niels Tørring, Ajay Kumar, Sven Olaf Skouby

**Affiliations:** ^1^Department of Obstetrics and Gynecology, Herlev Gentofte Hospital, Herlev, Denmark; ^2^Department of Translational Medicine, Lund University, Lund, Sweden; ^3^Department of Obstetrics and Gynecology, Skåne University Hospital, Malmø, Sweden; ^4^Reproductive Medicine Centre, Skanes University Hospital, Malmø, Sweden; ^5^Obsterics and Gynecology, Denmark and London Women's Clinic, Zealand University Hospital, London, United Kingdom; ^6^Laboratory of Reproductive Biology, Copenhagen University Hospital, Copenhagen, Denmark; ^7^Department of Clinical Biochemistry, Aarhus University Hospital, Aarhus, Denmark; ^8^Ansh Labs LLC, Medical Center Blvd, Webster, IA, United States

**Keywords:** anti mullerian hormone, antral follicle count, biomarkers, infertility counseling, female infertility

## Abstract

This study examined longitudinal, age-related and intra-individual variation in Anti-Müllerian Hormone (AMH) in regular menstruating women and correlated the hormonal levels to the antral follicle count (AFC). The impact of variations on an algorithm for calculation of follitropin-dose for ovarian stimulation were also tested. The study was carried out at a fertility clinic of a tertiary university hospital and had a prospective trial design. Twenty-six healthy women not receiving infertility treatment aged 22 to 50 years participated. Blood sampling for hormonal analysis was done every fifth day throughout three consecutive menstrual cycles, AFC was determined with 3-dimentional ultrasound and AMH measured by different assays from Beckman Coulter, Roche and Ansh Labs. Outcome measures were maximum and minimum difference in absolute and relative terms for each study subject during the test-period, coefficient of variation (Cv) for AMH for each cycle and cycle-day and correlation between AMH and AFC. The impact from variable AMH levels on an algorithm calculating follitrophin-delta dose in ovarian stimulation was explored. A significant longitudinal age-independent variation in AMH-levels and coefficient of variation in cycles and cycle days was found. A strong correlation between AMH-levels and AFC was confirmed and a case of significant divergence between assays was seen. Variations in AMH had a significant impact on an algorithm calculated dosage of gonadotrophins in ovarian stimulation. The finding of a substantial longitudinal variation in AMH question one recording being sufficient in quantifying gonadotrophins for ovarian stimulation, decision making and prognostication related to infertility treatment and counseling. Occasionally, commercial assays may fail to recognize specific AMH cleavage-products.

## Introduction

The serum concentration of Anti-Müllerian Hormone (AMH) has gained widespread clinical use as a surrogate marker for ovarian reserve. Currently, AMH measurements are used in human fertility counseling (1), to predict age of menopause (2), to diagnose polycystic ovarian syndrome (PCOS)(3, 4) and to predict response to ovarian stimulation (OS) (5, 6). As AMH levels may have major implications for clinical decisions on whether or not to proceed with IVF, to change to egg donation, to plan delay childbearing and attaining optimal ovarian stimulation during treatment, AMH measurements should be reliable and consistent.

The clinical use of AMH has to a large extent been facilitated by a reported relatively stable serum concentration during the menstrual cycle and an age-related decline bridging several decades until being exhausted at menopause. However, the interpretation of AMH results have previously been complicated by the use of different assay-standards and assay-characteristics (7–10). AMH undergoes proteolytic cleavage to become biologically active and additional proteolytic processing readily takes place (11). This processing, which may differ between individuals, exposes new antigenic sites which may affect measurements as well as AMH epitopes being masked by protein interaction in the circulation (12). This is confirmed by recent recognition of gender differences in AMH processing as western blot analysis of immature human granulosa cells exposed several other forms of AMH compared to those present in human fetal testicular tissue (11).

Serum levels of AMH are significantly associated with the number of antral follicles available for OS (13) and therefore AMH concentrations are widely used to predict high and low responders. A new recombinant follicle stimulating hormone preparation (rFSH) has recently been introduced to the market with an algorithm using serum level of AMH for the estimation of an appropriate starting dose of exogenous FSH in OS (14, 15).

In light of these findings and the fact that several recent studies have questioned the stability of AMH concentrations across the menstrual cycle (16) as well as one study reporting variation between cycles (17), the present study was initiated to evaluate serum AMH concentrations in two age matched cohorts of women during three consecutive cycles. Each blood sample was measured by three different AMH assays; the Beckman Coulter Gen II manual assay, the automated Roche Elecsys assay and the pico AMH (24/32) from Ansh Lab, and the variability was evaluated. Furthermore, a new algorithm launched for quantifying the starting dose of a new rFSH (Rekovelle®) for OS was tested, looking into the consequences of variable AMH values on the dosing dose.

## Subjects and methods

This prospective observational study was conducted at the Reproductive Medicine Centre, Skåne University Hospital Malmö, Sweden (blood-sampling and hormonal assaying), Herlev-Gentofte Hospital, University of Copenhagen, Denmark, Laboratory of Reproductive Biology (LRB), Copenhagen University Hospital, (Rigshospitalet), Denmark (computing and interpretation of data) and Aarhus University Hospital, Denmark (hormonal assaying). The Ethical Committee at Lund University, Sweden, approved the study.

### Study subjects

Between November 2011 and June 2012, healthy, regularly menstruating women aged 22–50 years were recruited, aged < 30 years using recruitment posters at the hospital directed toward hospital employees and medical or nursing students. Potential study subjects answered a standardized questionnaire concerning health, pregnancies, menstrual cycle length and received oral and written information before signing a consent form. Inclusion criteria were regular menstrual cycle in the range of 21–35 days, no actual use of tobacco or hormonal medication including oral contraceptives. Women with POCS were excluded.

In order to explore potentially age-related differences in AMH-variations, a total of 26 healthy non-smoking volunteer women, 16 below 30 years and 10 above 35 years fulfilling the criteria were subsequently initiated participation in the study. Two participants had 1 month's halt between cycle 2 and 3 and one women dropped out after fulfilling sampling in 2 cycles but remained in the study population. For 24 of the study subjects, measurements were performed in consecutive cycles.

### Blood sampling

The protocol included blood-sampling starting at menstrual cycle day 5 and continued every fifth day until the next menstrual bleeding where after the same procedure was repeated for two more cycles. Study subjects called the research team on the first day of the menstrual bleeding for initiation and planning of blood-sampling and vaginal ultrasound according to the schedule indicated above. Each blood sample, consisting of 10 mL blood, was drawn into vacuumed vials containing gel through a heparinized catheter inserted into a forearm vein. Within 2 h, the samples were centrifuged at 2,000 *g* for 10 min, and serum from each individual was divided into 3 vials before being stored at −20°C thus completing three frozen vial-lines from the same serum samples. When all samples from one study subject was completed, the vials were moved and stored at −80° until each of the samples were analyzed on three AMH ELISA platforms.

### Assays

AMH is secreted as a non-active homodimer precursor united by covalent bonds, which cleave by means of a proteolytic processing to a biological active associated dimer. Binding of the associated non-covalent complex to the receptor causes the N-terminal to dissociate into a non-active N-terminal (pro-region) and an active mature C-terminal (18). Detectable forms in serum would thereby theoretically be;

1. non-active AMH-precursor dimer (covalent).
2. active pro-mature dimer (associated).
3. non-active N-fragment pro-region.
4. active mature C-terminal.

However, a new proteolytic cleavage take place around the localization of amino acid 229 and also 451 (19, 20), giving rise to additional AMH-fragments like pro-mid-mature and mid mature fragments. Cleavage of larger AMH-sections, like the pro- and mature fragment, into sub-fragments may display new epitopes targeted by the antibodies or potentially blinding others, features which may impact on the ELISA-quantitation of the hormone. Probably, processing the molecule is probably individual and display time-related changes illustrated by the finding of different forms of the AMH-molecule across the menstrual cycle (9).

The samples were analyzed by three AMH ELISA assays at different occasions and locations. During the first round in 2012/2013, serum AMH was analyzed at Skåne University Hospital, Malmø, Sweden, using the Gen II manual kits from Beckman Coulter Inc., Marseille [30].

The lowest detectable level (LOD) distinguishable from zero with 95% confidence is 0.7 pmol/l. Coefficient of variation (%) of the Beckmann Coulter manual assay, calculated as standard deviation (SD)/mean) ^*^ 100, were 25% at 5.7 pmol/l and 12% at 52 pmol/l. All samples from one study subject were assayed in one run.

For the automated Roche Elecsys assay the similar calculations (%) were 1.9% at 6.3 pmol/L and 1.9% at 31.2 pmol/L The LOD of the assay was set to 0.5 pmol/l.

The inter-assay coefficient of variation (%) was determined by measuring daily replicates of controls in two levels in 36 consecutive workdays, with the result of 1.9% at both levels (6.3 and 31.2 pmol/l).

These two assays use 2 identical monoclonal antibodies directed against epitope regions located within the mature and pro-regions of the AMH molecule (21, 22).

During the third comparison, the samples originally assayed with Beckman Coulter 2012/2013 (Malmø) and refrozen were analyzed at Ansh Labs, Texas in 2016. Here, a new ELISA assay named pico AMH (24/32) with two monoclonal antibodies directed toward epitope regions in pro and mature regions of AMH was used. The antibodies used here are thus different compared to the one used in the Beckman Coulter and Elecsys Cobas assays. The LOD of the assay is 0.0086 pmol/l and total of 5.8% at 0.16 pmol/l and 4.4% at 2.7 pmol/L.

### 3D ultrasound images

Ultrasound analysis was performed using 4D-view™ software, version 9.1 (GE Medical systems, Zipf, Austria) with Sonography-based Automated Volume Calculation (SonoAVC™) software by one observer (LJ) and calculations were performed on multi-planar images showing the ovary in the longitudinal, transverse and coronal planes. SonoAVC software was used to calculate the number and size of antral follicles and average diameter were determined and listed according to their size. The SonoAVC report displays the automated measurements of the mean diameter (relaxed sphere diameter), maximum dimensions (x, y, z diameters) and volume of each object.

Most follicles, as hypo-echoic structures within a relatively hyper-echoic ovarian stroma, can be analyzed using SonoAVC software. However, to ensure that all follicles become recognized the volume of the ovary was finally examined manually in longitudinal and transverse planes to find follicles that had not been detected by the SonoAVC software, or had been incorrectly identified, and thereafter the follicle number was corrected.

The mean diameter and the number of follicles with a diameter of 2.0–10.0 mm were used for statistical analysis.

### Statistics

Coefficient of variation (Cv), calculated as standard deviation (SD)/mean, was used as measure to describe and compare variations between groups. SD was highly dependent on mean values whereas we saw lower dependence between Cv and mean value.

The distributions of Cv were inspected visually and by QQ-plots. Comparisons of Cv were performed using Repeated measures of ANOVA. Two participants with all values below detection limit in a cycle or at a cycle day were not included in comparisons of Cv (#2 and #8). Additionally one participant (#6) had low mean values of AMH including several measures below detection limit and high *Cv*-values. This Participant was included in calculations but to investigate whether the inclusion of this participant changed the results sensitivity analyses excluding this were performed.

Correlations between AMH and AFC-count were analyzed using Spearman Rank correlation. Confidence interval for these were calculated by a bootstrap method taking into account that each participant had several measures.

For calculating the effect of mean level AMH on the variation, one-way ANOVA was used.

All tests were two-sided. *P*-values below 0.05 were considered statistical significant. All statistics were performed using R version 3.3.3 (R Foundation of Statistical Computing, Vienna, Austria).

## Results

### Effect of age on the coefficient of variation

No statistical difference in variation expressed by Cv was found between the age-groups (Data not shown). As a consequence all study-subjects were subsequently treated as one group.

### Absolute and relative differences in levels of AMH during the study period for each study-subject

The minimum difference between the lowest and highest measured level of AMH measured among the study-subjects was found to be 3.9 pmol/L in absolute value during the study period for Beckman Coulter, 4.4 pmol/L for Elecsys Cobas and 0.5 pmol/L for the Ansh Labs assay (Table 1). The corresponding maximum differences were 33.0, 31.4, and 45.5 pmol/L, respectively. The minimum and maximum relative differences expressed in percentage for the study-subject with the lowest and highest changes over 3 cycles (% relative difference = highest- lowest value/lowest value^*^100) was found to be 60 and 446% for Beckman Coulter, 37 and 274% for Elecsys Cobas and 42 and 728% for Ansh Labs assay (Table 1).

**Table 1:** Absolute and relative differences was calculated in each woman of the total population.

	**Beckman coulter**	**Elecsys cobas**	**AnshLab**
Min diff (absolute value), pmol/L	3.9	4.4	0.5
Min diff, % (highest-lowest/ lowest * 100)	60	37	42
Max diff (absolute value), pmol/L	33.0	31.4	45.5
Max diff, %	446	274	728

*The table provides the difference recorded in the individual woman with the highest and lowest detected value*.

**Figure 1:**
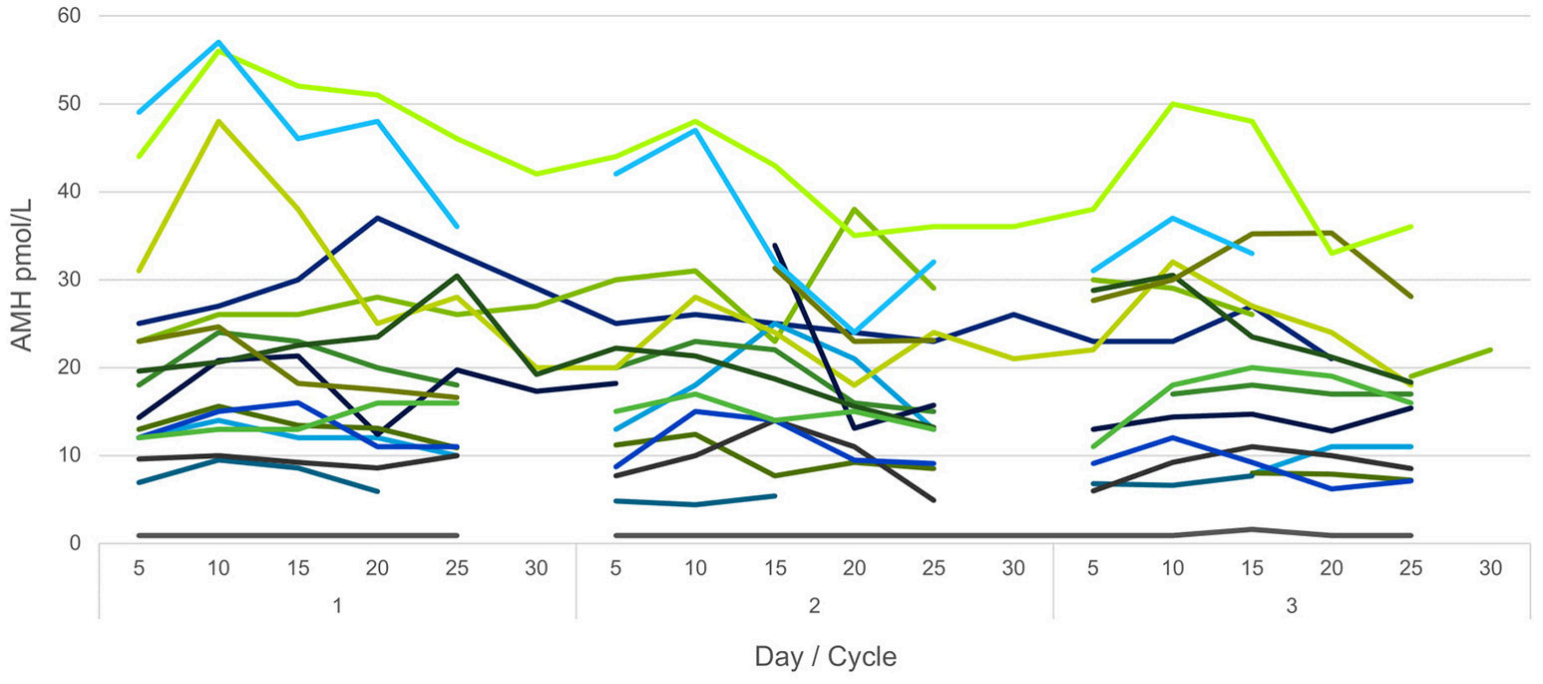
Intra-individual variation in AMH levels in the 16 women below 30 years throughout the study period. Assay: Beckman Coulter Gen II.

**Figure 2:**
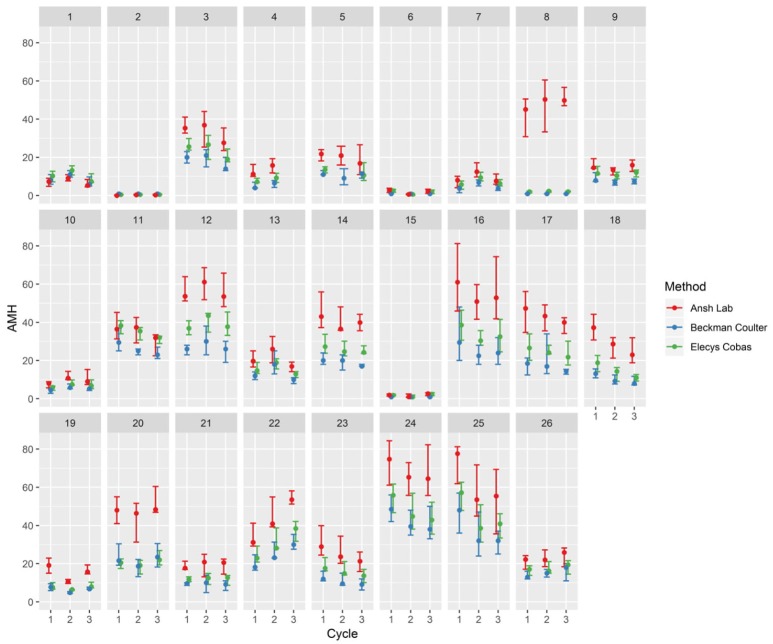
Median and range of AMH in three consecutive cycles with AMH measured cycle-day 5–10–15–20–25 at three analyzing platforms.

**Figure 3:**
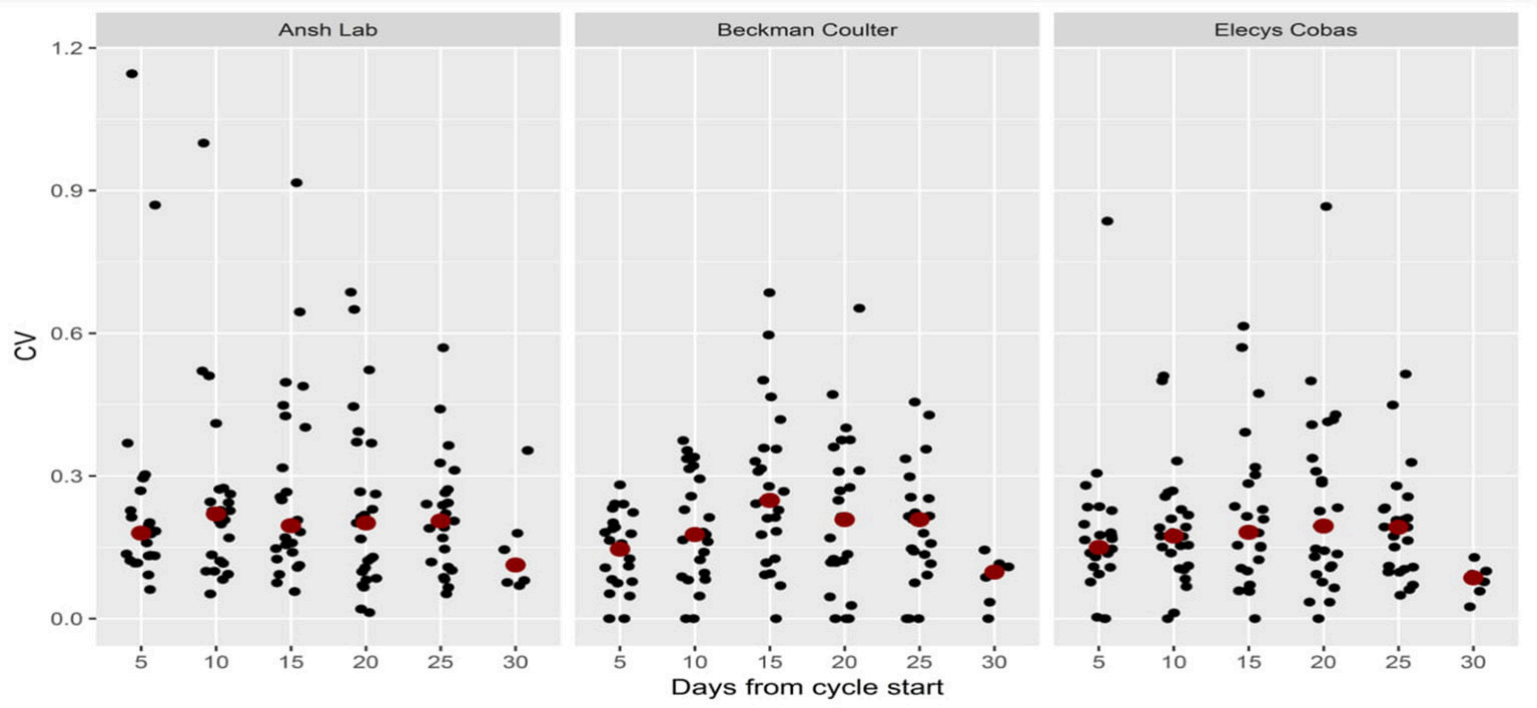
Coefficient of variation (CV) per cycleday in three assay-groups.

An overview of the intra-individual variation in AMH-levels throughout the study period for a younger group of the study population (below 30 years) is shown in Figure 1. The variation in all three assays (median and range) for cycles is shown in Figure 2 for women above 35 years and in Figure 3 for women below 30 years.

### Coefficient of variation (Cv)

**Figure 4:**
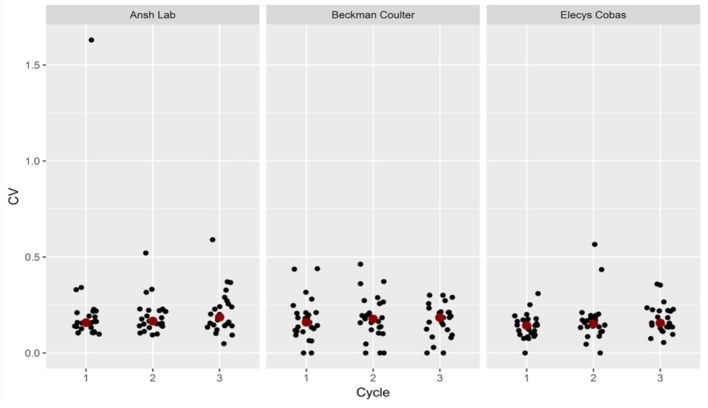
Coefficient of variation (CV) per cycle in three assay-groups.

**Figure 5:**
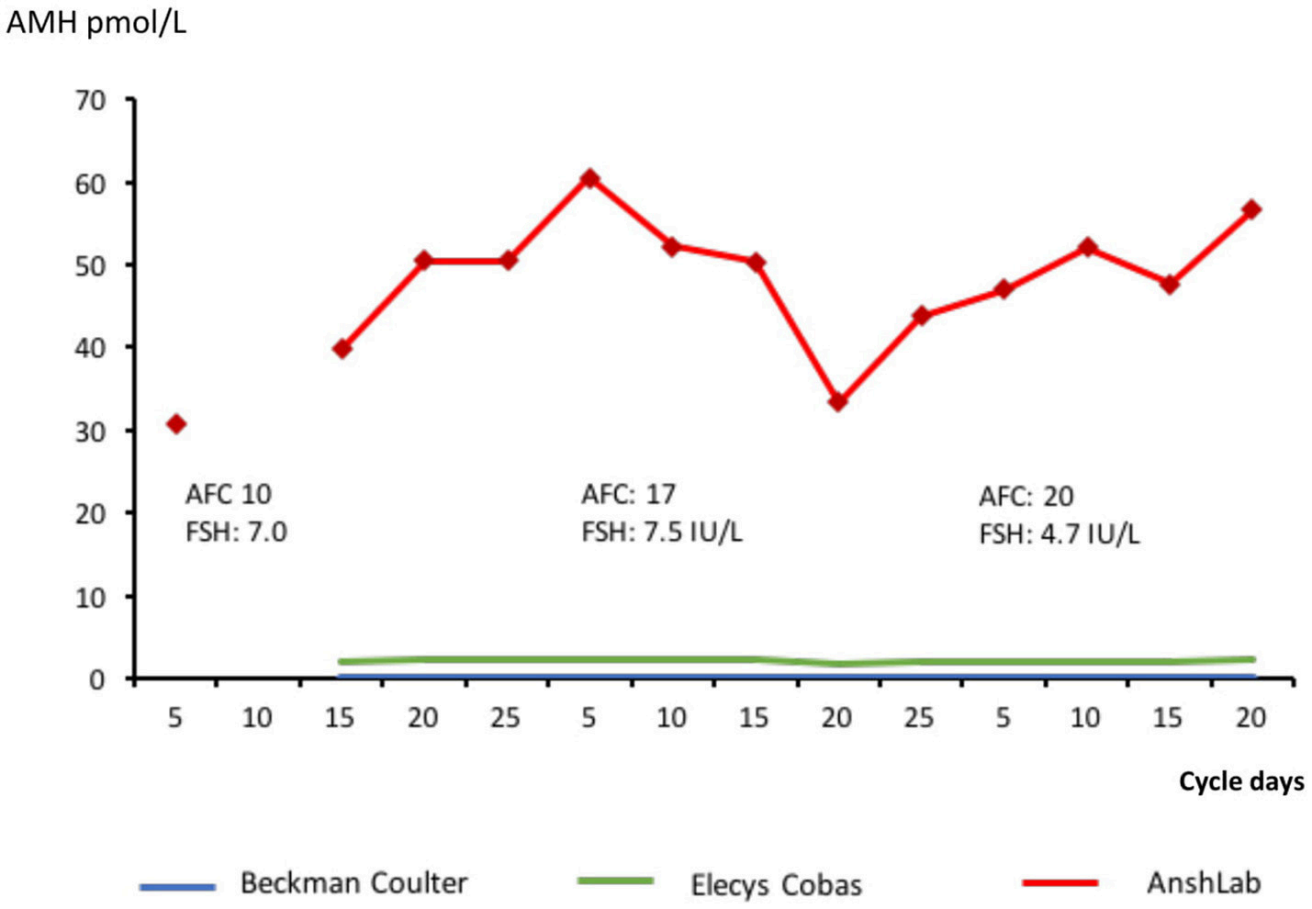
A case of significant discrepancy between assays. The Beckman Coulter assay was not able to detect AMH and all readings have the value zero. Antral follicle count and FSH recordings are added.

The individual variation in between cycles in the same patient was 0.0–0.46 for Beckman Coulter, 0.0–0.57 for Elecsys Cobas and 0.10–1.63 for Ansh Labs. The corresponding value for each cycleday within the same cycle were 0.0–0.69 for Beckman Coulter, 0.0–0.87 for Elecsys Cobas and 0.06–1.15 for Ansh lab (Table 2). The mean and median values for CV were similar indicating a symmetrical distribution (Table 2) with the highest variation found for the Beckman Coulter assay (Table 2). A graphical presentation of the individual variation for each cycleday is given in Figure 4 and pr. cycle in Figure 5. This displays graphically the huge dispersion in the recorded values in the same study-subject in the course of three menstrual cycles, as Cv is calculated from the equation standard deviation/mean.

**Table 2:** Coefficient of variation per cycle-day and within cycle measured in three consecutive cycles.

**Cycle day**	**Beckman coulter**	**COBAS**	**Ansh lab**	***P*-value between assays**
5	0.14 (0.08) *0.15 (0.00–0.28)*	0.18 (0.16) *0.16 (0.00–0.84)*	0.21 (0.16) *0.18 (0.06–0.87)*	0.11
10	0.19 (0.11) *0.18 (0.00–0.37)*	0.20 (0.12) *0.18 (0.01–0.51)*	0.22 (0.12) *0.21 (0.05–0.52)*	0.63
15	0.29 (0.16) *0.26 (0.07–0.69)*	0.23 (0.15) *0.18 (0.06–0.61)*	0.25 (0.16) *0.19 (0.06–0.64)*	0.016
20	0.23 (0.16) *0.21 (0.00–0.65)*	0.25 (0.19) *0.21 (0.03–0.87)*	0.22 (0.17) *0.18 (0.01–0.65)*	0.67
25	0.20 (0.12) *0.21 (0.00–0.46)*	0.20 (0.12) *0.19 (0.05–0.51)*	0.21 (0.13) *0.20 (0.05–0.57)*	0.90
30	0.10 (0.04) *0.11 (0.03–0.14)*	0.07 (0.03) *0.08 (0.02–0.10)*	0.11 (0.05) *0.08 (0.07–0.17)*	0.16
Within cycle-day *p*-value (cycleday 30 excluded with only 6 recordings available)	0.003	0.11	0.69	
**CV Between three individual cycles**				
**Cycle**	**Beckman coulter**	**COBAS**	**Ansh lab**	***P*****-value between assays**
1	0.18 (0.11) *0.17 (0.00–0.44)*	0.15 (0.06) *0.14 (0.07–0.31)*	0.17 (0.06) *0.16 (0.10–0.34)*	0.025
2	0.19 (0.11 *0.18 (0.00–0.46)*	0.18 (0.11) *0.16 (0.05–0.57)*	0.19 (0.09) *0.16 (0.09–0.52)*	0.99
3	0.18 (0.08) *0.18 (0.00–0.30)*	0.18 (0.08) *0.16 (0.05–0.36)*	0.20 (0.09) *0.17 (0.05–0.37)*	0.51
Within cycle *p*-value	0.70	0.10	0.31	

*Each cell shows values of CV as mean (SD) in the first row and in second roe in italics in median (range). Two participants were excluded from these analyses (#2, #8) due to immeasurable levels of AMH*.

Using Repeated measures ANOVA, differences in the Cv across the cycle days were evident. Subsequent analysis revealed these to be significant between measurements on cycle day five and fifteen. Testing differences in between cycles revealed significant altered variation in cycle one compared to cycle two and three. Sensitivity analyses excluding one participant with very low mean values and high Cv values did not change conclusions (data not shown).

### Effect of mean level AMH on the coefficient of variation

The study subjects were stratified according to mean level of AMH calculated from the Elecsys Cobas recordings, and divided in low AMH (< 10 pmol/L), medium AMH (10–20 pmol/L and high AMH (>20 pmol/L). The group with the lowest AMH recordings had a significant higher Cv tested with the Elecsys Cobas and AnsLab assay but not with the Beckman Coulter assay (Table 3).

**Table 3:** The effect of mean level AMH (Elecsys Cobas recordings) on the coefficient of variation.

	**Low (< 10 pmol/L)**	**Medium (10–20 pmol/L)**	**High (>20 pmol/L)**	***P***
Ansh lab	0.36 (0.14)	0.21 (0.05)	0.17 (0.04)	0.0006
Beckman coulter	0.28 (0.08)	0.25 (0.06)	0.21 (0.06)	0.15
Elecsys cobas	0.33 (0.16)	0.20 (0.05)	0.16 (0.04)	0.006

*Mean (SD). P-values by one-way ANOVA*.

### Correlation between AMH and antral follicle count

A strong correlation was found between AMH level and AFC-count on cycle day 5, *rs* = 0.83 for Elecsys Cobas, 0.84 for Beckman Coulter and 0.86 for Ansh Lab (Table 4).

**Table 4:** Spearman's Rank Correlation between AMH and AFC on menstrual cycle day 5.

	R_s_ (95% CI)^*^
Beckman coulter	0.83 (0.72–0.94)
Elecsys COBAS	0.83 (0.67–0.93)
AnshLab	0.85 (0.69–0.93)

* *Confidence limits calculated by bootstrapping*.

### A case of significant discrepancy between assays

One study subject displayed significant divergent readings between the assays, with immeasurable AMH with Beckman Coulter assay, values between 1.3 and 2.3 with Elecsys Cobas assay to 30.8–60.5 pmol/l for picoAMH ELISA assay from Ansh Labs (Figure 5). In all 3 cycles, her follicular phase estradiol and luteal phase progesterone indicated complete normal ovulatory cycles. Ultrasound detected antral follicle count at cycle day 5 of each cycle were; 10 (cycle 1) −20 (cycle 2) and 23 (cycle 3).

### The calculated effect of varying AMH levels on the follitropin delta- dose if treated according to algorithm

Table 5 shows the differences in follitrophin delta dose calculated from maximum and minimum recorded AMH values in the case of using a single AMH recording and bodyweight as argument in the dose-algorithm (14) of Rekovelle®. The calculated differences reveal no change in dose for women with a low ovarian reserve with a fixed dose to as much as 3.67 mcg (50 IU) (1 mcg = 13.7 IUI/L) in women with high ovarian reserve.

**Table 5:** The calculated effect of varying AMH levels on the follitrophin delta-dose if treated according to algorithm.

**Study subject**	**Age (ys)**	**Body-weight (kg)**	**AMH Cobas pmol/L max**	**AMH Cobas pmol/L min**	**Dose rFSH (mcg) max**	**Dose rFSH (mcg) min**	**Difference in dose rFSH (mcg) (max AMH–minAMH)**	**Difference in dose rFSH (IU/l) (max AMH–minAMH)**
1	41	64	16	7	12,0	12,0	0,0	0,0
2	50	63	1	1	12,0	12,0	0,0	0,0
3	40	80	32	18	12,0	9,66	2,34	31,9
4	39	50	11,6	6,8	12,00	12,00	0	0,0
5	46	70	17	8	12,0	12,0	0,00	0,0
6	45	57	3	0	12,0	12,0	0,0	0,0
7	41	65	12	3	12,0	12,0	0,0	0,0
8	42	80	2,4	1,3	12,0	12,0	0,0	0,0
9	37	55	15,3	8,7	12,0	12,00	0,0	0,0
10	42	59	9,9	3,5	12,0	12,0	0,0	0,0
11	25	64	40,9	28,9	7,66	6,33	1,33	18,1
12	29	65	45,4	33,1	7,00	6,66	0,34	4,6
13	25	65	20,9	11,1	12,00	9,66	2,34	31,9
14	29	70	33,7	22,4	10,66	7,66	3,00	40,9
15	27	75	3,1	0,7	12,0	12,0	0,0	0,0
16	25	62	46,3	24,5	8,0	6,33	1,67	22,8
17	26	58	33,9	17,6	10,0	6,33	3,67	50,0
18	27	66	22,6	9,1	12,0	9,33	2,67	36,4
19	27	57	10,3	6,0	12,0	12,0	0,0	0,0
20	22	69	26,9	14,6	12,0	9,00	3,00	40,9
21	26	62	14,9	8,7	12,0	11,66	0,34	4,6
22	25	83	42,1	20,8	12,0	8,33	3,67	50,0
23	26	61	23,2	10,1	12,0	8,33	3,67	50,0
24	28	63	61,7	35,5	7,00	6,33	0,67	9,1
25	24	69	62,6	31,3	8,33	7,00	1,33	18,1
26	28	58	21,6	13,9	12,0	8,66	3,34	45,5

## Discussion

The major finding of this study is a substantial overall intra-individual variation in AMH levels which to the best of our knowledge has not previously been reported with sample collection at 5 days intervals during three consecutive menstrual cycles taking only normally menstruating woman into account. The relative within-person biological variability ranged from 37 to 728% and by far exceeded the analytical variability of the three assays that ranged from 1.9 to 25% which clearly demonstrate a biological variation, which cannot be accounted for by assay variability.

AMH is generally considered a reliable and stable marker of ovarian function. Thus, in contrast to markers like FSH, no recommendations regarding a particular cycle day for optimal AMH assessment are currently recommended Furthermore, in the clinical context, distinct AMH cut off concentrations are often utilized to discriminate between normal and high or low values. Concentrations of AMH in a range of 1.4–9.0 pmol/L have been suggested to predict low response to hormonal stimulation (22–24), while levels as high as 48.9 pmol/L (25) and as low as 10.7 pmol/L (26) have been used to predict hyper response. Age-dependent AMH-cutoff levels have also been recommended as useful tools for predicting clear clinical outcome of assisted reproductive technologies (27). The results of the present study indicate that at least in normally menstruating women, over a period of three consecutive cycles, the measured AMH level may vary considerably. A recent randomized study (28) based on a published algorithm (29) reported no improvement in adding an AMH-reading for individualized hormone dosage compared to a conventional regimen in controlled hormonal stimulation for IVF, which reinforce this notion. The high variability in the coefficient of variation in cycles and cycle-days supports a more random regulation of the AMH-production most likely reflecting the effect of different factors regulating the number of follicles with diameters of around 5 to 8 millimeters that predominantly produce the AMH measured in circulation (13).

These data suggests the need to reevaluate the validity of founding the design of an individualized ovarian stimulation regimen on a single AMH measurement. Indeed, it is possible that excessive reliance on a single AMH measurement could expose the high responding patient to an increased risk of OHSS in case where AMH-based algorithm is used for calculating gonadotropin-dose for ovarian stimulation.

We found no difference in variation over time between the age-groups, and the former reported age-dependent pattern (23) of AMH-secretion could not be verified. The mean level of AMH had a significant impact on the coefficient of variation with highest Cv in the lowest group (AMH < 10 pmol/L) in the recordings obtained with the Ansh Lab and Elecsys Cobas assays. This effect was not seen in the Beckman Coulter assay which may be explained by a wide dispersion of recordings in women with low AMH, and unlike Ansh Lab and Elecsys Cobas displaying measurable but very low values in some study-subjects, the Beckman Coulter assay returned immeasurable values in the same women which recordings were excluded from statistical calculations (Table 3).

The Beckman-Coulter assay has been considered an insufficiently precise AMH measurement technique compared with the new automated Elecsys Cobas-assay (8, 24) although it uses identical monoclonal antibodies for measurement (25, 26). However, this study shows that the intra and inter-cycle variability was similar (Table 2). The third assay from Ansh labs used two different antibodies addressing other epitopes, but revealed the same variability over time in most situations. The observed AMH variability seems more likely to be explained by biologic variability. The highly significant correlation between the AMH-readings (Figure 5) in the three assays reinforces this conclusion. The level of AMH is different between the three assays, which could reflect different standards used for calibration alternatively the measure of different forms of AMH.

The importance of the assay used is underlined by the reported observations in one subject where both the Beckman Coulter and the Elecsys Cobas assays revealed close to immeasurable values of AMH. However, as measured by the picoAMH ELISA assay, her ovarian reserve appeared to be extremely high (40–60 pmol/l) (Figure 5). These findings were consistent with ultrasound measurement of a corresponding number of antral follicles at cycle day 5 of each of the 3 cycles (*n* = 10, 20, 23). Moreover, the magnitude of estradiol in the follicular phase and progesterone in the luteal phase indicated a normal ovarian function. This result reflects the differences that may occur when monoclonal antibodies recognizing different epitopes are used. In this case protein interactions, with for instance follistatin, may mask the epitopes recognized by the identical antibodies used in the Beckman Coulter and Elecsys Cobas assay, which has been reported to affect AMH signaling (12).

Similar discrepancies have been reported in women approaching menopause where more than 95% of the AMH in the circulation could be detected by the 24/32 (PicoAMH ELISA) and 24/37 (Ansh labs) while only 36% could be detected by Beckman Coulter gen II assay (27). This observation is probably due to the amount of complex cleavage products in serum from females.

The limitations of the study is a restricted number of study subjects with a wide dispersion in AMH-recordings which may influence the calculated variations. However, the number of repeated samples with an average of 15 measurements pr. individual throughout three consecutive menstrual cycles exceeds the number of measurements reported by earlier longitudinal studies. Additionally, blood samples from each individual study subject was collected and assayed in one run on three recognized platforms eliminating inter-assay bias described in earlier reports (28). Furthermore, we focused on normally menstruating women and not PCOS-women or patients undergoing infertility treatment. Still, there is no reason to believe that the relative intra-individual variation among subfertile women, in general, will differ from the cohort on women studies here. It should be emphasized that the Beckman Coulter assay used in this study was a manual assay which later has been replaced by automated technique displaying higher precision (29).

In conclusion, AMH provides considerable information on the ovarian reserve. However, a significant physiological intra-individual biological variation is present questioning the clinical validity of a single AMH-measurement in certain clinical settings. These may include important counseling connected to IVF-treatment or using AMH-based dosing algorithms in ovarian stimulation. As documented, commercial assays will in some individuals not be able to quantify their ovarian reserve due to existence of different AMH- forms or interaction with other proteins altering epitope exposure.

## Ethics statement

This study was carried out in accordance with the recommendations for Health and Biomedical Research. The protocol was approved by The Regional Ethical Review Board, Lund, Sweden. All subjects gave written informed consent in accordance with the Declaration of Helsinki.

## Author contributions

LB, CYA, and AG was responsible for the concept and design of the study and writing of manuscript. JT was responsible for recruitment and acquisition of blood samples. LJ performed ultrasound and participated in the writing of manuscript. MB, NM, and SOS participated in writing of manuscript. TK was responsible for statistics and writing of manuscript. NT and AK was responsible for assaying blood samples and writing of manuscript.

### Conflict of interest statement

The funders had no role in study design, data collection and analysis, decision to publish, or preparation of the manuscript. AK is employed by AnshLabs, which is a producer of one of the kits used in the study. He had no role in study design, data collection and analysis, decision to publish, or preparation of the manuscript, but performed blindly the analysis of samples with AnshLab's kit, as clearly stated in the manuscript. The remaining authors declare that the research was conducted in the absence of any commercial or financial relationships that could be construed as a potential conflict of interest.
